# Unraveling Alström syndrome: Homozygous mutation c.2729C>G in *ALMS1* gene across an extended family

**DOI:** 10.1002/mgg3.2314

**Published:** 2023-11-08

**Authors:** Salma A. S. Abosabie, Sara A. Abosabie, Jaber Alfaifi, Youssef A. Alqahtani, Ayed A. Shati, Najmah A. Alotaibi, Ohoud A. Alghamdi, Ghadi N. Alotaibi, Abdulrahman A. Baabdullah, Lama K. Kabrah, Naglaa M. Kamal, Mohammed A. M. Oshi, Enas A. A. Abdallah

**Affiliations:** ^1^ Faculty of Medicine Julius‐Maximilians‐Universität Würzburg Wurzburg Germany; ^2^ Faculty of Medicine Charité—Universitätsmedizin Berlin Berlin Germany; ^3^ Department of Child Health, College of Medicine University of Bisha Bisha Saudi Arabia; ^4^ Department of Child Health, College of Medicine King Khalid University Abha Saudi Arabia; ^5^ Department of Pediatrics Alhada Armed Forces Hospital Taif Saudi Arabia; ^6^ Faculty of Medicine Taif University Taif Saudi Arabia; ^7^ College of Medicine Umm Al‐Qura University Makkah Saudi Arabia; ^8^ Department of Pediatrics and Pediatric Hepatology, Kasralainy Faculty of Medicine Cairo Egypt; ^9^ Departement of Pediatrics Gaafar Ibnauf Children's Emergency Hospital Khartoum Sudan

**Keywords:** *ALMS1* gene, Alstrom syndrome

## Abstract

**Background:**

Alström syndrome (AS) represents an exceptionally rare genetic disorder characterized by a constellation of features including cardiomyopathy, progressive hearing and vision impairment, as well as obesity. This study seeks to elucidate the genetic underpinnings of this syndrome within the Saudi Arabian population.

**Methods:**

Employing an extended family cohort, we conducted an exhaustive molecular genetic assessment to delineate the presence of Alström syndrome. Additionally, we conducted an extensive review of existing literature from Saudi population to contextualize our findings within the broader understanding of the disorder in our country.

**Results:**

Within our studied extended family, we identified two individuals harboring the homozygous pathogenic mutation (c.2729C>G) in the *ALMS1* gene [NM_015120.4:c.2729C>G (p.Ser910*)]. Notably, carrier status was observed in the parents, whereas some siblings exhibited typical alleles while others were carriers of the mutation. Intriguingly, a review of the literature unveiled six distinct reports documenting a total of 20 Alström syndrome patients within the Saudi Arabian population, each presenting with distinct novel mutations.

**Conclusions:**

In cases featuring cardiomyopathy, obesity, and progressive hearing and vision loss, Alström syndrome merits inclusion within the differential diagnosis. To confirm the diagnosis, molecular genetic assessment of the *ALMS1* gene is imperative, offering definitive clarity amidst the complex clinical presentation. This investigation reinforces the importance of genetic scrutiny for precise diagnosis and highlights the unique genetic landscape of Alström syndrome within the Saudi Arabian population.

## Graphical abstract

Alström syndrome is a very rare genetic disorder that needs a high suspicion index in any patient with cardiomyopathy, obesity, and diminution of vision and hearing. Molecular genetic testing of the *ALMS1* gene uncovered a novel homozygous mutation (c.2729C>G) in an extended Saudi Family.

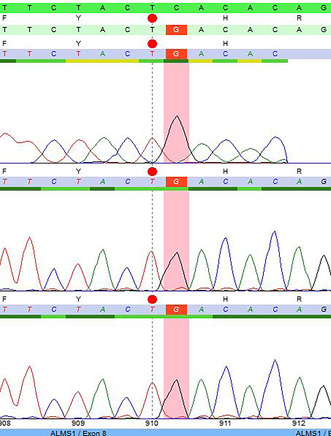

## 1 INTRODUCTION

Alström syndrome (AS), designated by OMIM as 606844, stands as an orphaned pediatric disorder notable for its distinct clinical hallmarks encompassing obesity, cardiomyopathy, and gradual progressive hearing and visual loss (Saadah et al., 2021). AS has a multisystemic domain, characterized by pronounced clinical heterogeneity in terms of onset, progression rates, and symptomatology (Khan et al., 2015). Foremost among these features is the relentless degeneration of retinal rods culminating in blindness, an affliction that ensnares approximately 90% of patients by the age of 16 (Khan et al., 2015). This condition is classified under the umbrella of ciliopathies, a group of genetic disorders that collectively impair the function of cilia in diverse human cell types, thereby leading to cellular dysfunction in the renal tubules, spermatozoa, retinal cells, and other cells ultimately coalescing into the composite features of ciliopathic syndrome (Khan et al., 2015).

The clinical milieu of AS is distinguished by a progressive sensorineural hearing loss, early‐onset obesity, and a corollary tendency toward insulin resistance leading to type 2 diabetes mellitus and hypertriglyceridemia. Adulthood unfurls a tableau marked by stunted stature, cardiomyopathy, hypogonadism, and incremental dysfunction of pulmonary, hepatic, and renal systems (Ozantürk et al., 2015).

At the genetic epicenter of AS resides the Alström syndrome (*ALMS1*) gene, the prodigious orchestrator of *ALMS1* protein synthesis. The genomic location of *ALMS1* exists on 2p13.1 (Ozantürk et al., 2015). Over 200 pathogenic mutations have been cataloged within *ALMS1*, predominantly constituting nonsense mutations, while also encompassing deletions, insertions, missense alterations, and translocations (Ozantürk et al., 2015). *ALMS1* protein was found to play a role in microtubule organization, intraflagellar trafficking, endosomal recycling, and cell cycle (Liu & Chen, 2022).

The therapeutic approach pivots around symptom management, aiming to enhance both quality of life and longevity (Choudhury et al., 2021). The syndrome's archival annals trace back to 1959, with its global case count surmounting 500 since its inaugural documentation, with no predilection for gender (Saadah et al., 2021). Only five reports have been released from the Kingdom of Saudi Arabia (Al‐Adsani & Gader, 2010; Aldahmesh et al., 2009; Bakar et al., 2017; Kamal et al., 2020; Saadah et al., 2021; Safieh et al., 2016).

We aim to study Alström syndrome and its genetics in an extended family which will help us to define AS genetics in the Saudi population.

## 2 PATIENTS AND METHODS

We conducted extended genetic testing encompassing 12 members of a family, prompted by the clinical diagnosis of an index case of Alström syndrome in a sibling. The index case was a 13‐year‐old Saudi male, who had been under the care of another hospital before presenting to us. The patient's medical history revealed a challenging course marked by dilated cardiomyopathy, refractory to medical interventions, and punctuated by recurrent admissions due to heart failure and concomitant chest infections.

Parents noticed that their child has progressive loss of vision and hearing. They sought medical advice and the patient received hearing aids and eyeglasses with little improvement in his hearing and vision capabilities.

On presentation to our hospital, the patient had a picture suggestive of heart failure (tachypnea, tachycardia, and enlarged tender liver) necessitating his hospitalization. His physical appearance further attested to his ailment, evident in his distressed countenance, debilitated physique (weight: 25.5 kg, length: 131 cm, both below the 3rd centile), and jaundiced hue.

The patient looked distressed, ill‐looking, underbuilt (weight was 25.5 kg, just below the 3rd centile, and length was 131 cm below the 3rd centile), and jaundiced. His cardiac examination revealed muffled first and second heart sounds with pansystolic murmur propagated all over the precordium.

His initial laboratory investigations included complete blood count, renal functions, blood glucose, and serum electrolytes, and yielded unremarkable results. However, his liver functions revealed elevated total bilirubin (83.7 mmol/L), direct bilirubin (64.9 mmol/L), aspartate transaminase (75 U/L), and alanine transaminase (50 U/L). Concomitantly, he had hypoalbuminemia (26 mg/dL) and prolonged prothrombin time (17 s) with hepatomegaly detected clinically and by abdominal ultrasound.

**FIGURE 1:**
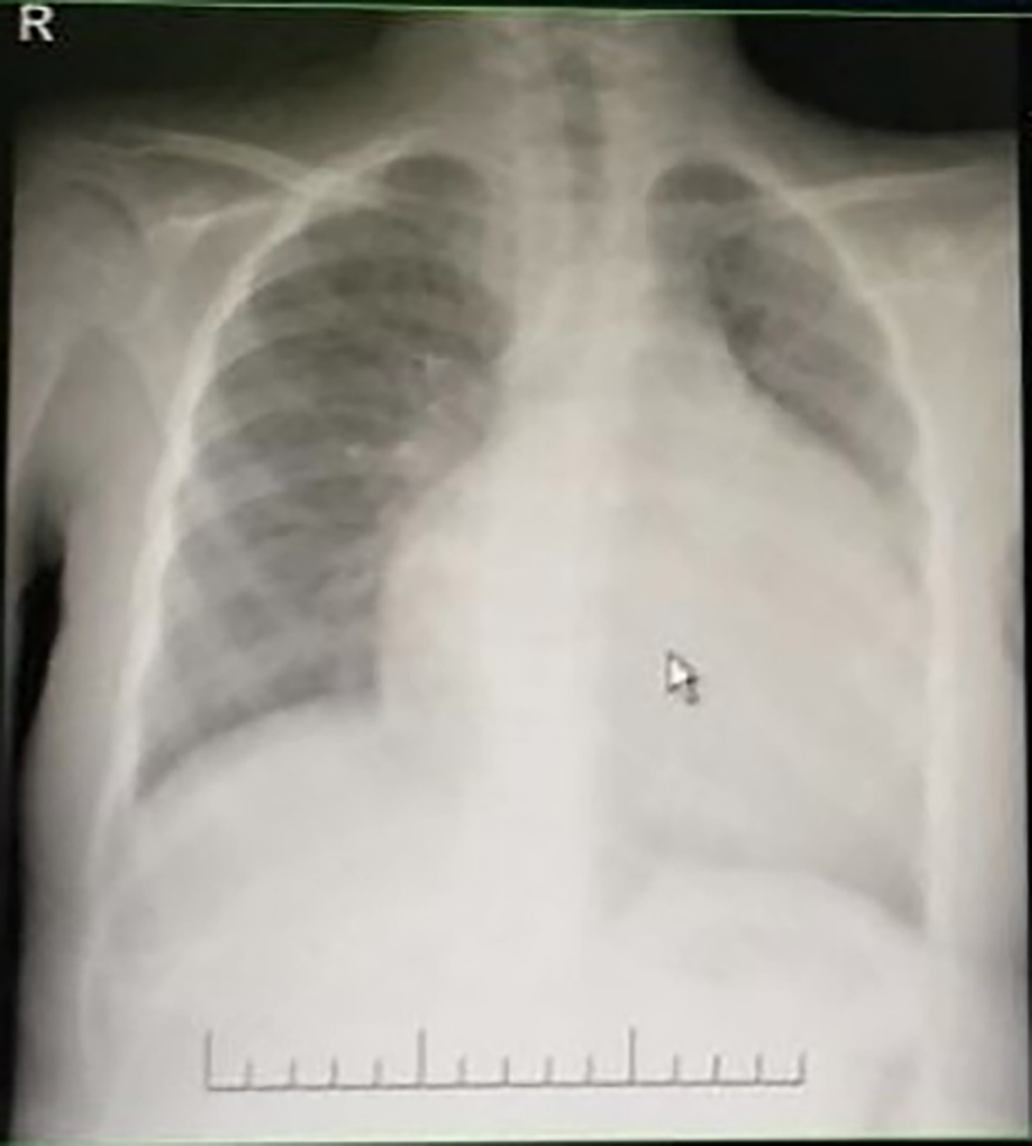
Chest x‐ray showing hugely dilated cardiomyopathy.

Radiological investigations revealed huge cardiomegaly and pulmonary congestion on chest radiography (Figure 1). Echocardiographic assessments disclosed dilated cardiomyopathy coupled with compromised cardiac function. Audiometric evaluations, along with auditory evoked potential studies, corroborated severe bilateral sensorineural hearing deficits.

In parallel, ophthalmological examinations unveiled substantial visual impairment, substantiated by visual evoked potential studies indicating bilateral retinal degeneration affecting both rods and cones.

Regarding the family history of the patient, he was born to a consanguineous parents (cousins) and he has a younger 6‐year‐old sibling who echoed analogous manifestations of auditory and visual impairment, albeit without concurrent clinical symptoms. Notably, eight additional siblings exhibited clinically unremarkable profiles.

In the realm of diagnostic precision, a multidisciplinary panel conducted an exhaustive reevaluation, converging on the triad of cardiomyopathy, auditory and visual impairments, and hepatic involvement reminiscent of Alström syndrome. Subsequent molecular analysis targeting the *ALMS1* gene was conducted to delineate the underlying genetic background of this intricate clinical presentation.

### 2.1 *ALMS* gene analysis (Figure 2)

**FIGURE 2:**
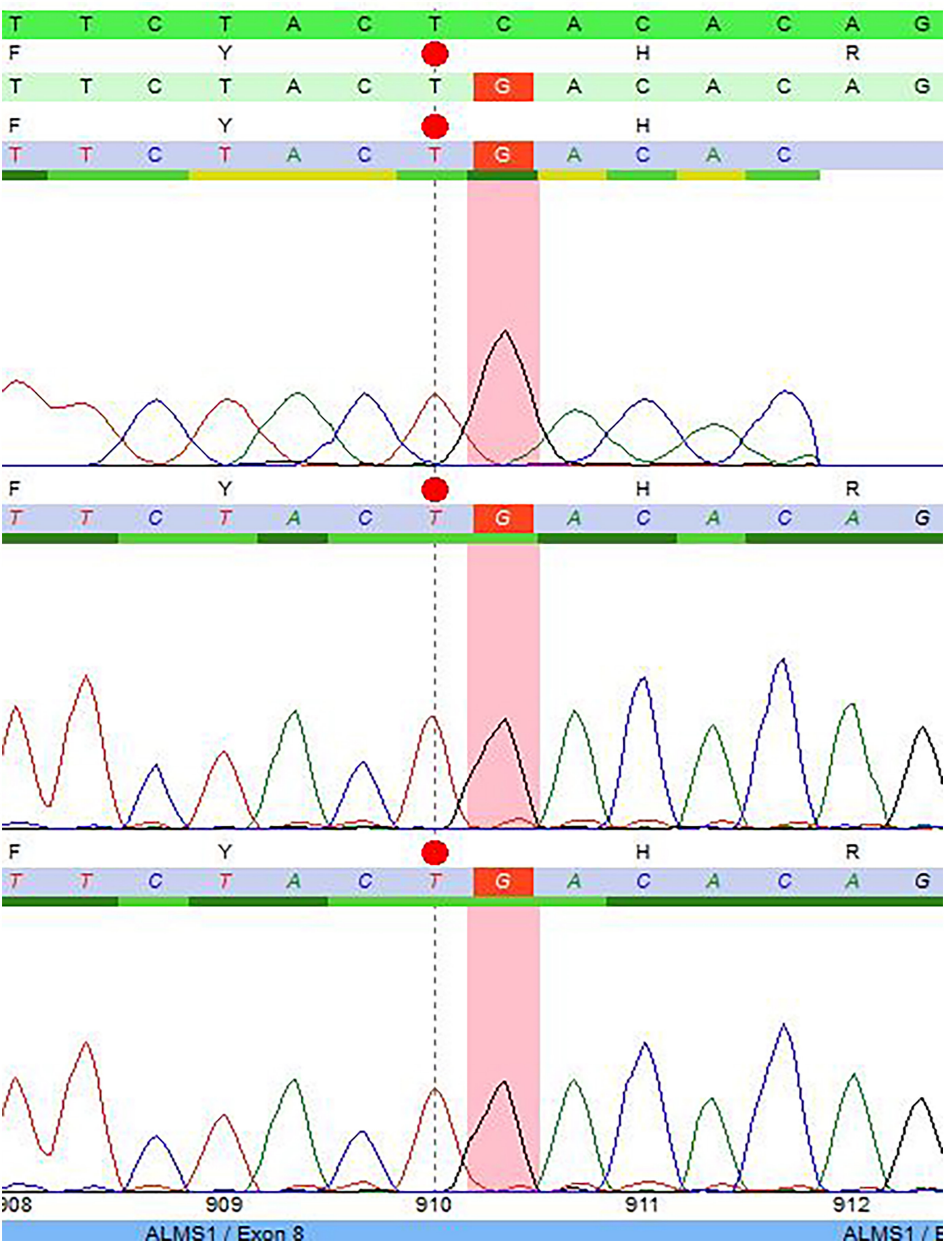
Chromatogram of the affected index case.

#### 2.1.1 *ALMS* gene analysis methods

Genomic DNA underwent screening for prevalent mutations within the *ALMS1* gene. Polymerase chain reaction (PCR) was employed to amplify exons 8, 10, and 16, alongside the exon‐intron boundaries of the *ALMS1* gene located on chromosome 2p13 (OMIM 606844). Subsequent direct sequencing facilitated the assessment of these target regions. Resultant sequence data were meticulously aligned with the reference sequence gene ID NM_015120.4. Notably, exons 8, 10, and 16 emerged as prominent mutational hotspots, encompassing more than 80% of the comprehensive spectrum of reported *ALMS1* mutations.

Supplementary analysis encompassing known ciliopathy‐associated genes was concurrently undertaken.

#### 2.1.2 *ALMS1* gene testing results

Sequence analysis detected a nucleotide alteration at position c.2729 within exon 8 of the *ALMS1* gene, in the form of a homozygous C‐to‐G substitution (c.2729C>G), as depicted in Figure 2. This nonsense exchange engenders a premature termination codon (p.Ser910*), instigating either the degradation of mRNA via nonsense‐mediated decay or truncation of the protein. Consequently, the detected variant is considered pathogenic.

Given the autosomal recessive inheritance pattern characterizing the disorder, the unequivocal identification of the aforementioned mutation in a homozygous state substantially substantiates the diagnosis of Alström syndrome.

To disentangle the distinction between pure homozygosity and compound heterozygosity, discerning between c.2729C>G (p.Ser910*) and a potentially large deletion encompassing this precise locus on the alternate allele, we carried genetic analysis of both parental genomes for the presence of this mutation as mentioned below.

### 2.2 Extended family study

The patient, his parents, and six siblings presented to us where the diagnosis was discussed. We explained that thorough history taking, clinical examination, and molecular genetic testing of the detected genetic mutation should be carried out to the whole family.

Following the definitive diagnosis of Alström syndrome in the index patient and in adherence to recommendations derived from the molecular genetic testing scientists, a telephonic communication was initiated with the parents of the patient. This contact aimed to arrange a comprehensive physical assessment involving all members of the extended family.

Subsequently, the patient, his parents, and six siblings partook in an in‐person consultation. During this session, family counseling was carried out and the confirmed diagnosis of AS in the index case was discussed with the parents. The imperative nature of an extensive evaluation, encompassing detailed medical history, comprehensive clinical examination, and molecular genetic testing targeting the identified genetic mutation for the extended family was thoroughly discussed with the parents who agreed about our plan.

### 2.3 History, physical examination, and investigations of the family

Thorough medical history inquiries coupled with exhaustive physical examinations yielded no noteworthy observations across the family, barring the younger sibling, a 6‐year‐old, who exhibited signs of diminution of both vision and hearing.

The clinical evaluation of this sibling demonstrated an optimal general health, stable vital signs, an elevated weight placing him above the 95th centile (denoting obesity), and a height aligned with the 50th centile.

Furthermore, jaundice was conspicuously absent. His cardiac assessment unveiled an ejection systolic murmur heard over the cardiac apex. Notably, no organomegaly was detected. Radiographic analysis, encompassing a chest x‐ray and abdominal ultrasound, were unremarkable while his echocardiography unveiled mild hypertrophic cardiomyopathy. The visual and auditory evoked potentials assessment highlighted bilateral rods and cones retinal degeneration and mild bilateral sensorineural hearing loss, respectively.

The constellation of these finding strongly pointed toward a highly plausible diagnosis of Alström syndrome within the 6‐year‐old sibling.

### 2.4 Molecular genetic testing of the detected *ALMS1* gene mutation (c.2729C>G) in the extended family

**FIGURE 3:**
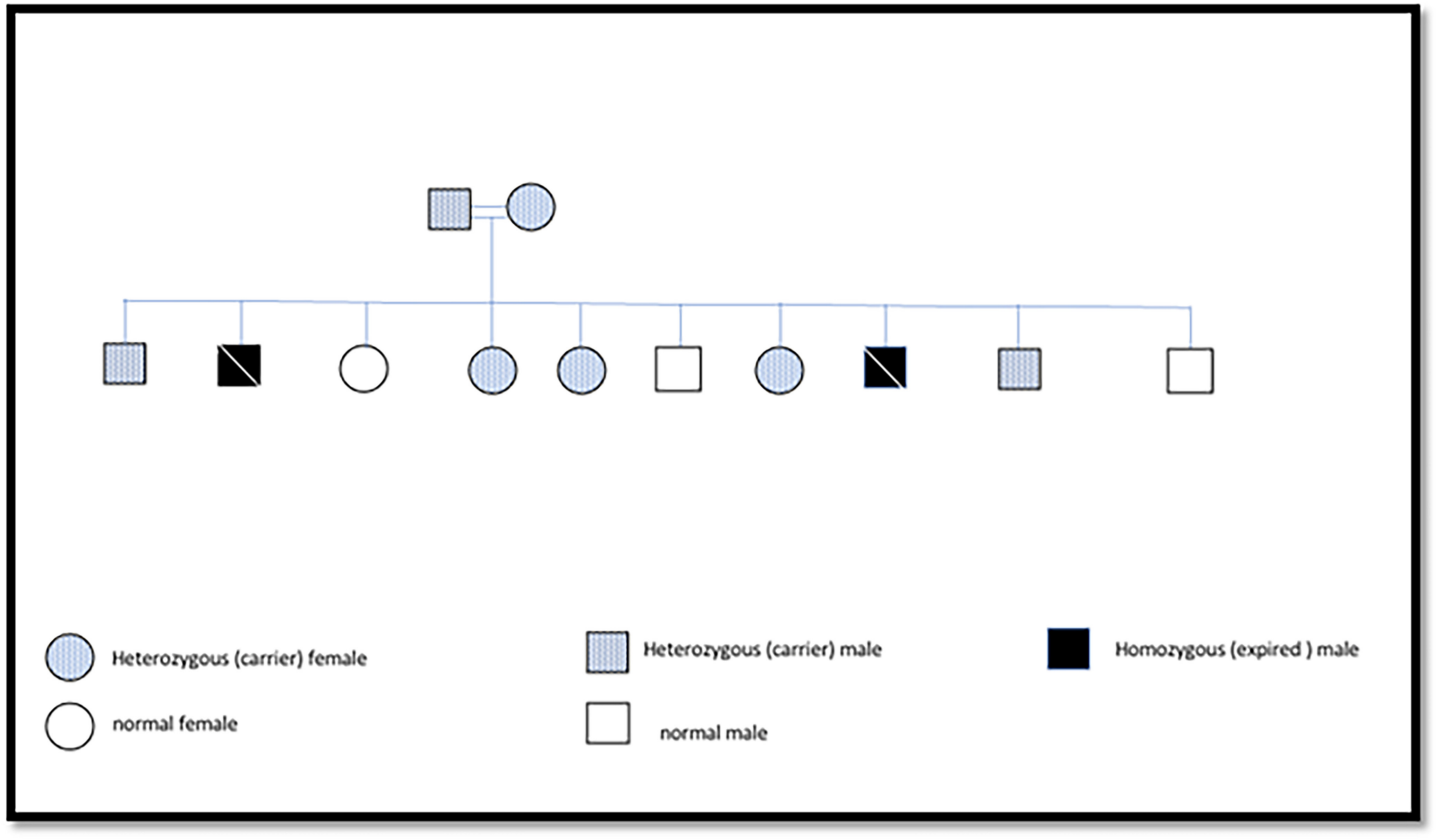
Family pedigree.

The entire extended family underwent molecular genetic testing to ascertain the presence of the c.2729C>G mutation within the *ALMS1* gene. A visual representation of the family pedigree is illustrated in Figure 3.

The genetic analysis conclusively confirmed the pathogenic mutation in a homozygous pattern within the 6‐year‐old sibling, thus substantiating the diagnosis of Alström syndrome. Both parents and three siblings demonstrated the status of carriers, displaying the pathogenic mutation in a heterozygous configuration while two siblings were negative (normal).

#### 2.4.1 Family counseling and therapeutic regimen

Family members were extensively counseled, receiving comprehensive insights into the disease's nature, its inheritance pattern, and the potential implications for future pregnancies.

In the absence of a definitive cure or preventive strategies for Alström syndrome, the current therapeutic approach emphasizes symptom management and enhancement of overall quality of life (Choudhury et al., 2021). To this end, a multidisciplinary care regimen was meticulously devised for the two affected siblings. The collaborative care plan involves regular consultations with specialists encompassing cardiology, ophthalmology, audiology, gastroenterology, endocrinology, pediatrics, and dietary consultation.

#### 2.4.2 Outcome and follow‐up

Tragically, the proband succumbed to refractory heart failure stemming from severe dilated cardiomyopathy at the age of 14. Conversely, the other affected sibling, presently 12 years old, demonstrates favorable general and cardiac conditions. While progressive hearing and vision loss remain evident. He is still obese and did not cross his weight centile to a higher one. He developed acanthosis nigricans, but he did not develop diabetes or liver complications.

Throughout a 6‐year follow‐up period, the family had three additional siblings. Comprehensive mutation testing revealed that two siblings, one male and one female, harbored the mutation in a heterozygous state, designating them as carriers. The third male sibling, however, tested negative, denoting a non‐affected status.

## 3 DISCUSSION

Alström syndrome, is characterized by multisystem involvement and a wide spectrum of clinical manifestations. The inheritance pattern of AS follows an autosomal recessive pattern, resulting in a distribution of outcomes with 25% affected by the disease, 25% normal, and 50% carriers during each pregnancy (Paisey et al., 2003). The identification of a pathogenic mutation in the *ALMS1* gene within a family member necessitates the comprehensive screening of close relatives. Recommendations for at‐risk relative testing, prenatal screening, and preimplantation genetic testing have been put forth, ensuring early detection and appropriate counseling (Paisey et al., 2003).

AS is a complex disorder affecting various organ systems. Among its hallmark features, multiple endocrine dysfunctions, particularly type 2 diabetes mellitus, and dilated cardiomyopathy (Mahamid et al., 2013). In addition to the endocrine and cardiac involvements, AS is characterized by a constellation of symptoms that can present at different stages of life. These include progressive sensory impairments, such as hearing and vision loss, which often lead to significant disability. Of note, 90% of patients experience blindness by the age of 16 due to progressive retinal degeneration (Khan et al., 2015).

The identification of biallelic pathogenic mutations in the *ALMS1* gene serves as a cornerstone in confirming the diagnosis and understanding the underlying pathophysiological mechanisms (Paisey et al., 2003). The global landscape of AS has been studied extensively, and several studies have shed light on its varied genetic and clinical manifestations. Marshall and colleagues performed a comprehensive analysis of AS patients worldwide, summarizing phenotypes and genotypes (see File S1) (Marshall et al., 2015). This collaborative effort underscores the international significance of AS research, revealing a wide spectrum of mutations that contribute to the syndrome's diverse clinical presentations.

We carried a similar task on the Saudi population where we reviewed English literature for the previously documented Saudi Arabian patients of AS and summarized their phenotypes and genotypes in Table 1 (Al‐Adsani & Gader, 2010; Aldahmesh et al., 2009; Bakar et al., 2017; Kamal et al., 2020; Saadah et al., 2021; Safieh et al., 2016). Within this subset of Saudi Arabian cases, our research group has contributed significantly, reporting five families with a total of 12 AS patients, with different novel mutations (Table 1) (Bakar et al., 2017; Kamal et al., 2020; Saadah et al., 2021).

**TABLE 1:** Reported patients of Alström syndrome from Saudi Arabia.

Patients	Type of mutation	Clinical picture	Reference
6 patients from 2 families	Rare ALMS1, 3′‐splice‐site acceptor (c.11873‐2 A>T) variant, which skips entire exon‐19 and shortens the protein by 80 amino acid	Cardiomyopathy progressive hearing loss blindness	Saadah et al. (2021)
1 patient	ALMS1 mutation c.12154_12166del (p.Arg4052Glyfs*2) in homozygous state	diabetes mellitus horizontal nystagmus obesity acanthosis nigricans sensorineural hearing loss poor vision, poor color vision, retinitis pigmentosa	Bakar et al. (2017)
5 patients from 2 families	ALMS for family A mutant gene: T376S in exon 5 and S909* in exon 8 Family B mutant: an R2721* in exon 10 Those novel mutation for Alström syndrome Will creates unstable protein, which eventually undergoes intracellular degradation	Family A: loss of visual acuity horizontal nystagmus sensorineural hearing loss photophobia obesity polyphagia acanthosis nigricans cardiomegaly Family B: loss of visual acuity horizontal nystagmus sensorineural hearing loss photophobia obesity acanthosis nigricans cardiomegaly mild cholestasis mild to moderate mental disability	Kamal et al. (2020)
5 patients from different families [Aldahmesh et al. (2009)]			
A‐ 8 years old boy	Homozygous mutation in ALMS1 locus Nonsense mutation c.5534C>G in exon 8 (S908X)	congenital nystagmus low vision sunken eyes Photophobia	Aldahmesh et al. (2009)
B‐ 2‐year‐old boy	Homozygous mutation ALMS1 locus Frameshift mutation also in exon 8, was a four base‐pair deletion (c.5981delCAGA) resulting in premature truncation at position 1992	congenital nystagmus low vision sunken eyes dilated cardiomyopathy photophobia	Aldahmesh et al. (2009)
C‐ 2‐year‐old boy	Homozygous mutation in ALMS1 locus Nonsense mutation c.8275C>T in exon 10, resulted in truncation of ALMS1 at codon 2720 (R2720X)	congenital nystagmus low vision dilated cardiomyopathy occasional head nodding retinal dystrophy	Aldahmesh et al. (2009)
D‐ morbidly obese 10 year‐old girl	Homozygous mutation in ALMS1 locus Splice‐site mutation that fully abolishes the consensus acceptor site resulting in complete skipping of exon 19 with consequent frameshift	short stature acanthosis nigricans deepset eyes retinal dystrophy	Aldahmesh et al. (2009)
E‐ 6 year old morbidly obese	Homozygozity by descent was confirmed at the ALMS1 locus Splice‐site mutation that fully abolishes the consensus acceptor site resulting in complete skipping of exon 19 with consequent frameshift	short stature acanthosis nigricans deepset eyes retinal dystrophy	Aldahmesh et al. (2009)
2 patients from 2 different families	Patient 1: Direct sequencing of the coding sequence of GUCY2D revealed a missense mutation affecting a highly conserved position (c.743C>T; p.S248L) Patient 2: Mutation screening of candidates genes revealed a pathogenic mutation in ALMS1 gene (c.8441C>A, p.S2814*)	Patient 1: oculodigital sign bilateral enophthalmos marked nystagmus Patient 2: photophobia short and stubby fingers hearing loss mid‐facial hypoplasia bilateral enophthalmos insulin dependent diabetes	Safieh et al. (2016)
34 years old male	ALMS1 2p1 mutations, located on exon 16 (40%), exon 10 (23%), exon 8 (21%), exon 6, 12, 17, 18	insulin‐resistant diabetes hypertension blindness primary infertility (hypogonadism) sensorineural deafness renal impairment hepatic dysfunction retinitis pigmentosa	Al‐Adsani and Gader (2010)

Saadah et al. identified a rare exon 19 skipping mutation in the *ALMS1* gene in unrelated Saudi families (Saadah et al., 2021), while the affected exon in this report is exon 8, emphasizing the genetic diversity in the Saudi population. Bakar et al. reported a novel mutation in a Saudi girl with insulin‐resistant diabetes, emphasizing the importance of early detection (Bakar et al., 2017). Kamal et al. contributed significantly by identifying *ALMS1* missense and stop gain mutations in familial AS patients (Kamal et al., 2020). Aldahmesh et al. demonstrated the allelic heterogeneity of AS in inbred Saudi populations, indicating the diverse genetic landscape (Aldahmesh et al., 2009). Safieh et al. added to this understanding by identifying novel mutations in Saudi patients with congenital retinal dystrophy (Safieh et al., 2016). Al‐Adsani et al. highlighted the co‐occurrence of diabetes mellitus and retinitis pigmentosa, shedding light on the intricate relationship between AS and metabolic disorders (Al‐Adsani & Gader, 2010). On the other hand, Khan et al. highlighted the significance of ophthalmic features in undiagnosed AS children (Khan et al., 2015).

Ozantürk et al. conducted a comprehensive analysis of AS in Turkish kindreds, underscoring the variability in clinical presentations (Ozantürk et al., 2015). Liu and Chen utilized exome sequencing to establish an accurate diagnosis of AS (Liu & Chen, 2022), a powerful tool that enhances diagnostic precision. The heterogeneous nature of AS was further exemplified by Choudhury et al., who reviewed AS as a rare monogenic ciliopathy (Choudhury et al., 2021). Such reviews underscore the complexity of AS and its clinical implications, promoting a comprehensive understanding of its diverse manifestations.

Furthermore, the genetic heterogeneity observed within Alström syndrome is mirrored in the clinical variability of its presentation. Mahamid et al. documented significant intrafamilial variability in disease progression among siblings, emphasizing the need for a nuanced understanding of genotype–phenotype correlations (Mahamid et al., 2013). They reported two siblings who initially presented during infancy with severe dilated cardiomyopathy. On follow‐up, while the older sibling demonstrated marked improvement upon discontinuation of medical therapy for dilated cardiomyopathy, the younger sibling's condition deteriorated despite appropriate medical intervention. This observation underscores the lack of genotype–phenotype correlations within Alström syndrome, exemplified by these siblings carrying the *ALMS1* gene variant NM_015120.4:c.2729C>G (p.Ser910*) while manifesting distinct phenotypes (Mahamid et al., 2013). This observation aligns with the broader genetic landscape of AS, where diverse mutations can lead to distinct clinical outcomes.

This is similar to our report, despite both siblings having identical mutation, the elder child (the index case) showed rapid progressive deterioration of his dilated cardiomyopathy with failure of medical management and death, while his younger affected sibling is running a more favorable course with mild hypertrophic cardiomyopathy.

Wang et al. expanded the disease spectrum by highlighting Alström syndrome as a potential differential diagnosis for Leber Congenital Amaurosis (LCA), a severe retinal dystrophy (Wang et al., 2011). This underscores the importance of considering AS in the context of early‐onset retinal disorders, expanding the differential diagnostic possibilities, and guiding appropriate clinical management.

Our study further contributes to the realm of Alström syndrome genetics in Saudi Arabia and globally by detailing two siblings harboring the homozygous pathogenic nonsense mutation, c.2729C>G, within exon 8 of the *ALMS1* gene. This mutation, which results in a premature termination codon and subsequent degradation of mRNA or protein truncation, adds to our understanding of the genetic underpinnings of AS.

The case of the patient and his sibling, diagnosed after 13 and 6 years, respectively, serves as a poignant reminder of the diagnostic challenges associated with Alström syndrome. Clinicians must maintain heightened vigilance for AS, particularly in patients presenting with cardiomyopathy, hearing and vision loss, obesity, and hepatic or endocrine manifestations.

Our study also highlights the importance of family‐wide molecular genetic testing, as evidenced by the identification of carriers among siblings. This underscores the necessity of identifying carriers for informed family planning and counseling.

Despite advancements, AS remains a clinical challenge with no specific cure. The management approach is currently symptom‐based, necessitating multidisciplinary care.

In conclusion, our study contributes to the evolving landscape of AS genetics, emphasizing its clinical heterogeneity, genetic diversity, and the necessity of comprehensive genetic testing for accurate diagnosis and informed management. While we mourn the loss of one sibling to refractory heart failure, our findings provide valuable insights for future research and enhance our understanding of AS within the Saudi population.

## AUTHOR CONTRIBUTIONS

All authors contributed substantially to writing the manuscript, reviewing the literature, the concept and design, acquisition, and interpretation of data; drafting the article, revising it critically for important intellectual content; and final approval of the version to be published.

## FUNDING INFORMATION

No funds were available for the current research.

## CONFLICT OF INTEREST STATEMENT

All authors declare no competing interests related to the study.

## ETHICS STATEMENT

The study was approved by the local research and ethical committee.

## CONSENT FOR PARTICIPATION AND FOR PUBLICATION

Parents signed written informed consents for their participation and for participation of their children in the current study and for publication of the current original research article.

## Supporting information


Table S1

## Data Availability

All data and materials related to the study are included in the current manuscript.
