# Supplementary figures and images for: Unraveling Alström syndrome: Homozygous mutation c.2729C>G in ALMS1 gene across an extended family
Source: Mol Genet Genomic Med. 2023 Nov 8;12(1):e2314. doi: 10.1002/mgg3.2314 (PMC10767606; doi:10.1002/mgg3.2314)

**Table 1. 109 Novel *ALMS1* Mutations**


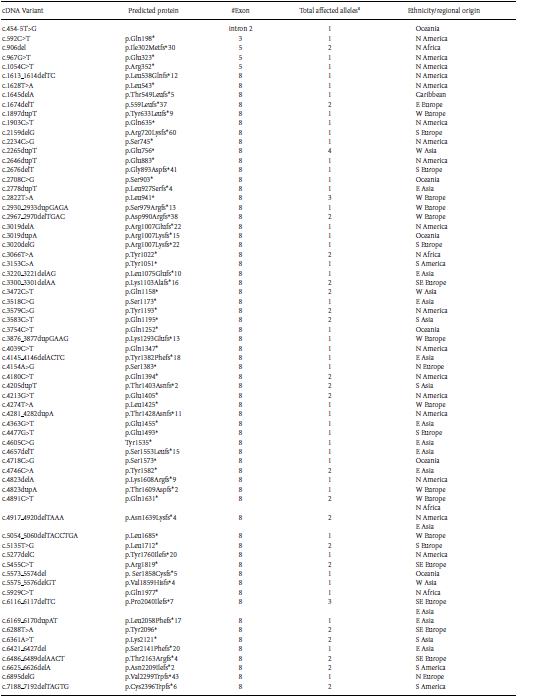


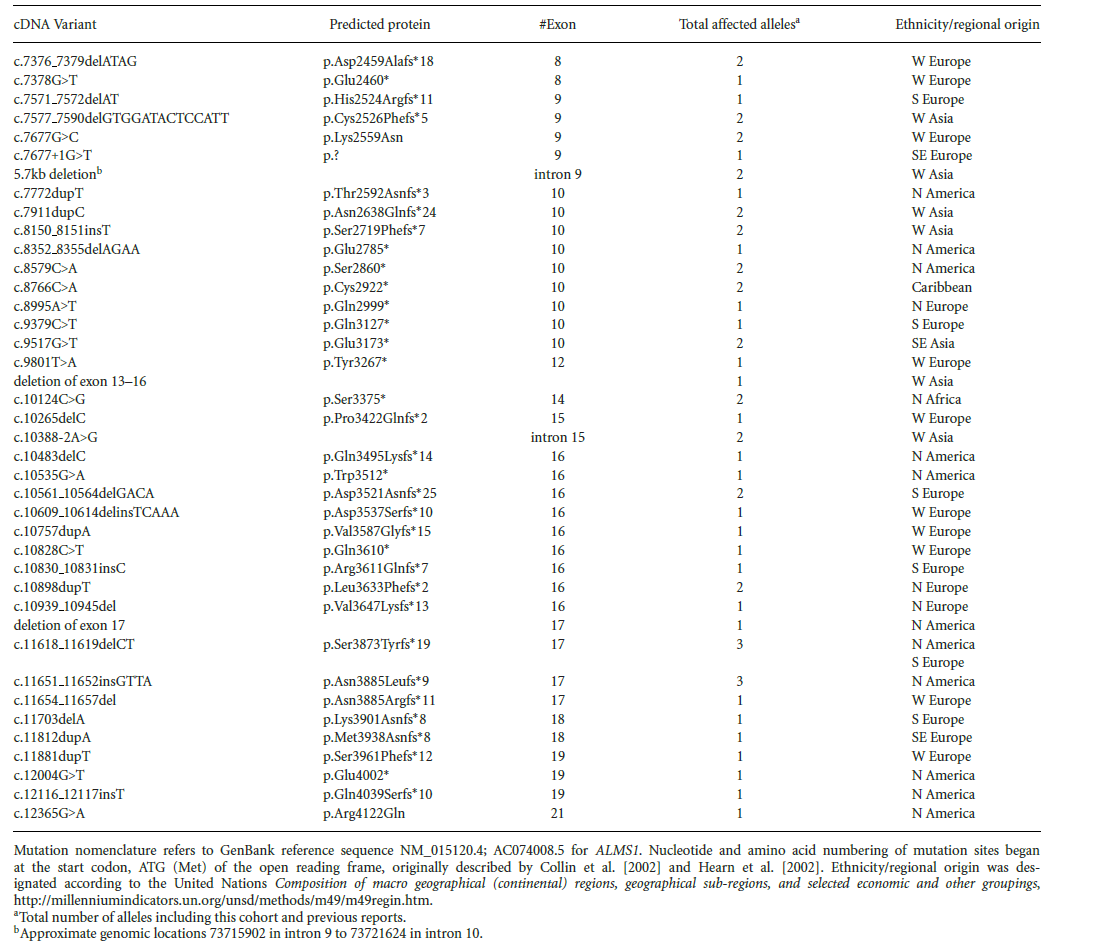


Table reproduced with permission (Marshall)

Supplement: Supplementary file 1 — Table S1 [file MGG3-12-e2314-s001.docx]
